# Supplementary material for: Mildew Resistance Locus O Genes CsMLO1 and CsMLO2 Are Negative Modulators of the Cucumis sativus Defense Response to Corynespora cassiicola
Source: Int J Mol Sci. 2019 Sep 26;20(19):4793. doi: 10.3390/ijms20194793 (PMC6801717; doi:10.3390/ijms20194793)
Supplement: Supplementary file 1 [file ijms-20-04793-s001.pdf]

Table S1 The disease index analysis in Jinyou 38 and Xintaimici in cucumber

Cucumber cultivars	Numbers	Days post-inoculation (dpi)	Disease index
Jinyou 38	77	7	14.85
Xintaimici	75	7	72.33

Note: High resistance (HR), 0＜DI≤15; Moderate resistance (R), 15＜DI≤35; Resistance(MR), 35＜DI≤55; Susceptible (S), 55＜DI≤75; High susceptible (HS), DI＞75.

Table S2 The primers of qRT-RCR used in this study

**Analysis**	**Primer name**	**Sequence(5′-3′)**
CsPR1-1a for qRT-RCR in transgenic cucumber	*CsPR1-1a* -F	GGCAGCCCAGACTTCTCAGC
	CsPR1-1a -R	GCATCTCACTTTGGCACATCCTA
CsPR2 for qRT-RCR in transgenic cucumber	*CsPR2*-F	AAGACAACATCCTCAACTTTGC
	CsPR2-R	CTGCTGAAAGAATTCCAGTGTG
CsPR3 for qRT-RCR in transgenic cucumber	*CsPR3*-F	CATTTCTCTCATCCTTTGGCAG
	CsPR3-R	GATATCGAAATCAACGCCATCC
CsPYL2 for qRT-RCR in transgenic cucumber	*CsPYL2*-F	CAGAAATCATTGATGGACGACC
	CsPYL2-R	TGAAACATCAGCAAGTGACTTG
CsPP2C2 for qRT-RCR in transgenic cucumber	*CsPP2C2*-F	TATTCCCGATCCAGAAGTCATG
	CsPP2C2-R	CAAGCATTGAGAGGTAATCTGC
CsSnRK2.2 for qRT-RCR in transgenic cucumber	*CsSnRK2.2*-F	GCAGATCCAGAGACGAGAATAA
	CsSnRK2.2-R	TCAATACTCTGTGTTGCCTCAT
CsABI5 for qRT-RCR in transgenic cucumber	*CsABI5*-F	GAATCGCCATTACAGTCTGAAC
	CsABI5-R	AACTCATCCAATGTGAGTGAGT
CsRbohD for qRT-RCR in transgenic cucumber	*CsRbohD*-F	GCGTAACATTACTGGTGATTCC
	CsRbohD-R	ATTCTTCCATCAGCATCCGTAT
CsRbohF for qRT-RCR in transgenic cucumber	*CsRbohF*-F	ATCGGACGATATTACACAGCTT
	CsRbohF-R	ACAATCACCAGAACGAATACCT
Cucumber *CsActin* gene for and qRT-PCR	Actin-F	TCGTGCTGGATTCTGGTG
	Actin-R	GGCAGTGGTGGTGAACAT

Table S3 List of primers used in the study

**Analysis**	**Primer name**	**Sequence(5′-3′)**
Sequencing of the cDNA of *CsMLO1*	*CsMLO1*-F	GATATCATGGCGGGGGCAGCCGGTGGCAA
	CsMLO1-R	GCGGCCGCTCATTCAACTCTATCAAATGAAAAGTC
Sequencing of the cDNA of *CsMLO2*	*CsMLO2*-F	GGATCCATGGCTGAATGTGGAACAGAG
	CsMLO2-R	CTCGAGTCATTTGGCAAATGAGAAGTCTGAG
qRT-PCR for *CsMLO1*	*RTCsMLO1*-F	ATGCGTTGGCTAGAGCTAAGATG
	RTCsMLO1-R	AGGTGTCTCTTGCAAACCTAAACC
qRT-PCR for *CsMLO2*	*RTCsMLO2*-F	GTCATTCACCTCACTGGAAAGTGG
	RTCsMLO2-R	AGTGACAGCATCTTGGCCTATCG
CsMLO1-silencing vector	*TRVCsMLO1*-F	GAATTCGTGGCAGAGGCCCTTCGCAAC
	TRVCsMLO1-R	GAGCTC TCATTCAACTCTATCAAATGA
CsMLO2-silencing vector	*TRVCsMLO2*-F	gtgagtaaggttaccgaattcCACCACTCAGCCAAGAAG
	TRVCsMLO2-R	ggcctcgagacgcgtgagctcTCATTTGGCAAATGAGAA
CsMLO1-overexpression vector	*LucCsMLO1*-F	tctgagctctctagaATGGCGGGGGCAGCCGGTGG
	LucCsMLO1-R	cataagcttctgcagTTCAACTCTATCAAATGAAA
CsMLO2-overexpression vector	*LucCsMLO2*-F	ctgagctctcagaATGGCTGAATGTGGAACAGA
	LucCsMLO2-R	cataagcttctgcagTTTGGCAAATGAGAAGTCTG
Chimeric primer for *CsMLO1*-nLuc	*CsMLO1-nLuc*-F	ACAACGATTCGCCCTCTCCATCTC
	CsMLO1-nLuc-R	CCTCGATATGTGCATCTGTAAAAGC
Chimeric primer for *CsMLO2*-nLuc	*CsMLO2-nLuc*-F	GTTGCCTCCTTCTTCACACCATAGC
	CsMLO2-nLuc-R	CCTCGATATGTGCATCTGTAAAGC

ATGGCGGGGGCAGCCGGTGGCAAGTCGCTGGAGCAAACACCGACATGGGCCGTTGCCGTTGTTTGCTTTGTTTTGCTCGTCATCTCTATT 90

M A G A A G G K S L E Q T P T W A V A V V C F V L L V I S I 30

TTCATCGAATATAGTCTCCATCTTATCGGACATTGGCTAAAGAAGAGACACAAACGGGCGTTGTTTGAAGCATTAGAGAAGATCAAATCA 180

F I E Y S L H L I G H W L K K R H K R A L F E A L E K I K S 60

GAGCTTATGTTATTGGGGTTTATATCATTGCTACTAACGGTGGGGCAAGGACCAATAACGGAGATATGTATTCCACAACATGTAGCTGCA 270

E L M L L G F I S L L L T V G Q G P I T E I C I P Q H V A A 90

ACGTGGCATCCATGTACAAAGGAAAGAGAAGATGAGATGAACAAAGAGGTGGAGAAATCTGTGGAACATTTGGGTCTTAATCGCCGGAGA 360

T W H P C T K E R E D E M N K E V E K S V E H L G L N R R R 120

CTCCTTCATCTCCTCGGAAATGGTGAAAGTTTCCGGCGGAGTTTGGCCGCTGCGGGAGGAGAGGATAAATGTGCCGCCAAGGGTAAAGCT 450

L L H L L G N G E S F R R S L A A A G G E D K C A A K G K A 150

TCCTTTATTTCAGCAGATGGAATTCATCAACTTCATATCTTCATTTTTGTGTTGGCTGTTTTTCATGTTTTGTATTGTGTTCTAACTTAT 540

S F I S A D G I H Q L H I F I F V L A V F H V L Y C V L T Y 180

GCGTTGGCTAGAGCTAAGATGAGGAGTTGGAAAACATGGGAAAAAGAGACCAAAACTGCTGAATACCAATTCTCACATGATCCAGAGAGG 630

A L A R A K M R S W K T W E K E T K T A E Y Q F S H D P E R 210

TTTAGGTTTGCAAGAGACACCTCATTTGGGAGAAGACATTTGAGCTTTTGGACCAAAAATCCTGCCTTGATGTGGATCGTTTGTTTCTTC 720

F R F A R D T S F G R R H L S F W T K N P A L M W I V C F F 240

AGACAATTTGTAAGATCTGTTCCAAAAGTTGATTACTTGACATTAAGACATGGGTTTATAATGGCACATTTAGCACCTCAAAGTCATACA 810

R Q F V R S V P K V D Y L T L R H G F I M A H L A P Q S H T 270

CAATTTGATTTTCAAAAATACATTAATAGATCCCTTGAAGAAGACTTCAAAGTTGTTGTGGGAATCAGCCCACCAATTTGGTTCTTTGCT 900

Q F D F Q K Y I N R S L E E D F K V V V G I S P P I W F F A 300

GTTCTATTTCTCCTCTCAAACACTCACGGTTGGAGGGCGTATCTATGGCTGCCATTCATCCCACTAATCATTTTGCTGTTGATTGGAACA 990

V L F L L S N T H G W R A Y L W L P F I P L I I L L L I G T 330

AAATTGCAAGTGATCATAACGAAAATGGCACTAAGAATACAAGAAAGAGGTGAAGTAGTGAAGGGCGTGCCGGTGGTGGAGCCTGGCGAT 1080

K L Q V I I T K M A L R I Q E R G E V V K G V P V V E P G D 360

GACCTCTTTTGGTTTAATCGACCTCGCCTTATTCTTTATCTCATCAACTTTGTTCTCTTTCAAAATGCCTTCCAAGTTGCCTTCTTTGCT 1170

D L F W F N R P R L I L Y L I N F V L F Q N A F Q V A F F A 390

TGGACTTGGTATGAGTTTGGGTTGAATTCTTGCTTCCATGAGCATATAGAAGATGTGGTGATCAGAATTTCTATGGGGGTGCTTGTACAA 1260

W T W Y E F G L N S C F H E H I E D V V I R I S M G V L V Q 420

ATCCTTTGCAGTTATGTTACTCTTCCTCTTTATGCACTAGTCACTCAGATGGGTTCAACAATGAAGCCAACTATATTCAATGAGAGAGTG 1350

I L C S Y V T L P L Y A L V T Q M G S T M K P T I F N E R V 450

GCAGAGGCCCTTCGCAATTGGTACCACTCGGCTCGAAAGCACATCAAACACAACCGCGGTTCGGTCACTCCAATGTCGAGCCGACCCGCC 1440

A E A L R N W Y H S A R K H I K H N R G S V T P M S S R P A 480

ACCCCGACTCACAGCATGTCACCTGTCCACCTTCTCCGACACTACAAGAGTGAAGTCGATAGCTTCCACACCTCACCGAGAAGGTCACCG 1530

T P T H S M S P V H L L R H Y K S E V D S F H T S P R R S P 510

TTCGACACCGATCGTTGGGACAACGATTCGCCCTCTCCATCTCGCCATGTTGATGGTTCGTCTTCGTCACAACCCCACGTTGAGATGGGA 1620

F D T D R W D N D S P S P S R H V D G S S S S Q P H V E M G 540

GGTTATGAAAAAGATCCCGTTGAATCAAGTTCGTCTCAAGTTGATCCGGTTCAACCATCTCGAAACCGCAATCAACATGAGATTCATATT 1710

G Y E K D P V E S S S S Q V D P V Q P S R N R N Q H E I H I 570

GGAGGCCCCAAAGACTTTTCATTTGATAGAGTTGAA**TGA** 1749

G G P K D F S F D R V E * 582

Figure S1 Nucleotide and deduced amino acid sequences of cucumber *CsMLO1* cDNA encoding an MLO homolog protein. The deduced amino acid sequences are given below the nucleotide sequences. The transcriptional start site is shown in bold type and the termination codon is marked by an asterisk (*). The amino acids shown in a solid box reveal a domain of the MLO-related protein, a member of the plant integral membrane proteins.

```
ATGGCTGAATGTGGAACAGAGCAGCGTACTTTGGAAGATACCTCAACTTGGGCTGTTGCGGTTGTTTGTTTTTTCTTGGTTGTTATTTCA   90
M  A  E  C  G  T  E  Q  R  T  L  E  D  T  S  T  W  A  V  A  V  V  C  F  F  L  V  V  I  S      30
ATCTTCATTGAACATGTCATTCACCTCACTGGAAAGTGGCTGGAGAAAAGGCACAAGCCAGCTCTTGTTGAAGCTCTAGAAAAGGTTAAA   180
I  F  I  E  H  V  I  H  L  T  G  K  W  L  E  K  R  H  K  P  A  L  V  E  A  L  E  K  V  K      60
GCAGAGCTTATGCTATTGGGATTCATATCCCTACTTCTAACGATAGGCCAAGATGCTGTCACTCAAATTTGTGTTTCGAAAGAGCTTGCA   270
A  E  L  M  L  L  G  F  I  S  L  L  L  T  I  G  Q  D  A  V  T  Q  I  C  V  S  K  E  L  A      90
GCAACTTGGCTTCCCTGTGCAGCAAGAGCTAAAACAGGAGTAAAAGTTGCGAAGAACAGTCGTCTTAGACTTCTTGAATTTTTAGATCCT   360
A  T  W  L  P  C  A  A  R  A  K  T  G  V  K  V  A  K  N  S  R  L  R  L  L  E  F  L  D  P      120
GACTATGGTTCGAGGCGTATTTTAGCCTCGAAAGGAGATGATGCATGCGCTAAGAGGGGCCAACTCGCTTTCGTGTCGGCATATGGAATC   450
D  Y  G  S  R  R  I  L  A  S  K  G  D  D  A  C  A  K  R  G  Q  L  A  F  V  S  A  Y  G  I      150
CATCAGCTCCATATTTTCATCTTCGTATTGGCTGTCTTCCATGTCCTATACTGCATCATAACTTTGGCTTTTGGCAGAACGAAGATGAGC   540
H  Q  L  H  I  F  I  F  V  L  A  V  F  H  V  L  Y  C  I  I  T  L  A  F  G  R  T  K  M  S      180
AAATGGAAGGCCTGGGAGGATGAAACCAAGACAATTGAATACCAGTACTATAATGATCCAGCAAGATTTAGATTTGCTAGAGATACTACG   630
K  W  K  A  W  E  D  E  T  K  T  I  E  Y  Q  Y  Y  N  D  P  A  R  F  R  F  A  R  D  T  T      210
TTTGGACGCCGACACTTGAGCTTCTGGAGTCGTACACCAATTTCCCTCTGGATTGTTTGTTTCTTCAAACAGTTCTTTGGATCAGTTACC   720
F  G  R  R  H  L  S  F  W  S  R  T  P  I  S  L  W  I  V  C  F  F  K  Q  F  F  G  S  V  T      240
AAGGTTGATTACATGACACTGAGACATGGATTCATCGTTGCACATCTTGCACCCGGAAGTGAAGTAAAATTTGATTTCCACAAATACATT   810
K  V  D  Y  M  T  L  R  H  G  F  I  V  A  H  L  A  P  G  S  E  V  K  F  D  F  H  K  Y  I      270
AGCAGATCTCTGGAAGACGACTTTAAAGTTGTTGTGGGGATTAGTCCCGCAATGTGGCTATTTGCTGTTCTCTTCATCCTAACCAATACA   900
S  R  S  L  E  D  D  F  K  V  V  V  G  I  S  P  A  M  W  L  F  A  V  L  F  I  L  T  N  T      300
AATGGGTGGTATTCATATCTATGGCTGCCTTTCATCTCCTTAATTATAATTCTATTGGTGGGAACAAAGCTCCATGTTATTATAACTCAT   990
N  G  W  Y  S  Y  L  W  L  P  F  I  S  L  I  I  I  L  L  V  G  T  K  L  H  V  I  I  T  H      330
ATGGGATTGACAATTCAAGAAAGGGGTCATGTTGTGAAGGGTGTTCCGGTCGTTCAGCCTCGGGATGACCTGTTTTGGTTTGGACGTCCA   1080
M  G  L  T  I  Q  E  R  G  H  V  V  K  G  V  P  V  V  Q  P  R  D  D  L  F  W  F  G  R  P      360
CAACTTATTCTCTTCCTGATCCACTTTGTTCTCTTTATGAATGCATTTCAGCTTGCCTTCTTTGCTTGGACCACTTATGCATTTAAGTGG   1170
Q  L  I  L  F  L  I  H  F  V  L  F  M  N  A  F  Q  L  A  F  F  A  W  T  T  Y  A  F  K  W      390
ATGGGTTGTTTCCATCAGCGAGTTGAAGATATTGTCATCAGACTCTCAATGGGGGTTATCATACAAGTTCTCTGCAGTTATGTCACACTC   1260
M  G  C  F  H  Q  R  V  E  D  I  V  I  R  L  S  M  G  V  I  I  Q  V  L  C  S  Y  V  T  L      420
CCACTCTATGCTTTGGTTACTCAGATGGGCTCTAACATGAGACCAACCATTTTCAACGACCGAGTGGCAACGGCATTGAAGAACTGGCAC   1350
P  L  Y  A  L  V  T  Q  M  G  S  N  M  R  P  T  I  F  N  D  R  V  A  T  A  L  K  N  W  H      450
CACTCCGCCAAGAAGAACATGAAGCAGCACCGCAACCCAGACAGTACCTCACCATTCTCAAGCAGGCCAGCTACTCCAACTCACGGCATG   1440
H  S  A  K  K  N  M  K  Q  H  R  N  P  D  S  T  S  P  F  S  S  R  P  A  T  P  T  H  G  M      480
TCTCCTATTCACCTTCTGCACAAACATCAGCATGGCAGCACATCTCCCAGGCTATCCGATGCCGAACCCGATCGTTGGGAAGAGTTGCCT   1530
S  P  I  H  L  L  H  K  H  Q  H  G  S  T  S  P  R  L  S  D  A  E  P  D  R  W  E  E  L  P      510
CCTTCTTCACACCATAGTAGAGCCCCCCATCATGATAATCATCAAGATCAACAAGAACAATCTGAGACAATAATTAGAGAACAGGAGATG   1620
P  S  S  H  H  S  R  A  P  H  H  D  N  H  Q  D  Q  Q  E  Q  S  E  T  I  I  R  E  Q  E  M      540
ACAGTTCAAGGACCAAGTTCAAGTGAAACCGGTTCCATAACACGTCCTGCTCGCCCTCATCAGGAAATCACTAGGACTCCATCAGACTTC   1710
T  V  Q  G  P  S  S  S  E  T  G  S  I  T  R  P  A  R  P  H  Q  E  I  T  R  T  P  S  D  F      570
TCATTTGCCAAATGA                                                                              1725
S  F  A  K  *                                                                                574
```

Figure S2 Nucleotide and deduced amino acid sequences of cucumber *CsMLO2* cDNA encoding an MLO homolog protein. The deduced amino acid sequences are given below the nucleotide sequences. The transcriptional start site is shown in bold type and the termination codon is marked by an asterisk (*). The amino acids shown in a solid box reveal a domain of the MLO-related protein, a member of the plant integral membrane proteins.

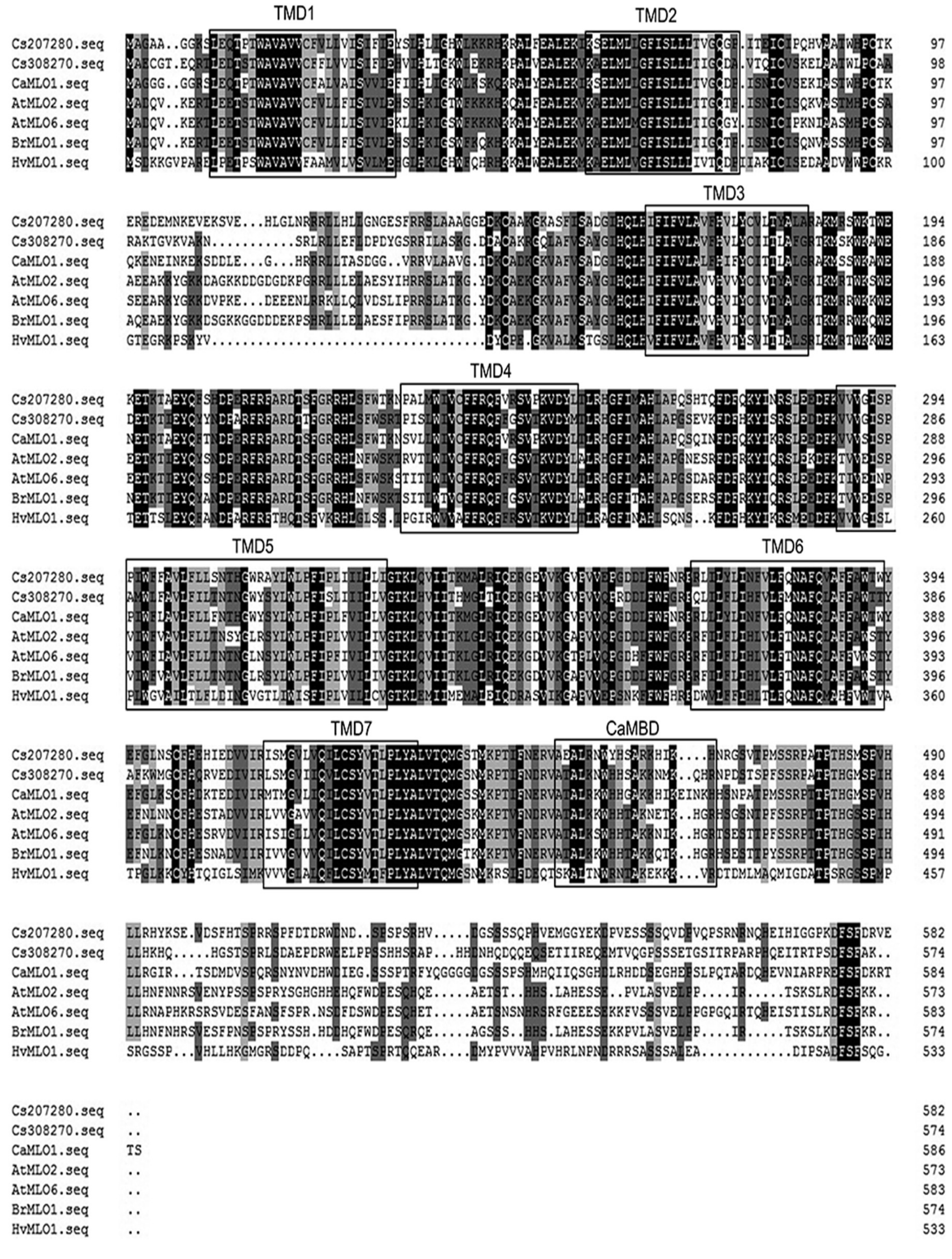

Figure.S3 Sequence analysis to *CsMLO1* and *CsMLO2* in cucumber. Comparison of the deduced amino acid sequence of *Cs207280* (*CsMLO1*) and *Cs308270* (*CsMLO2*) cDNA with other plant MLOs from pepper (*Capsicum annuum*, *MLO1*, accession no. AAX31277), Arabidopsis (*Arabidopsis thaliana*, *MLO2* and *MLO6*, accession no. Q9SXB6 and Q94KB7), turnip (*Brassica rapa*, *MLO1*, accession no. AAX77014) and barley (*Hordeum vulgare* subsp. *vulgare*, *MLO1*, accession no. P93766). The conserved seven transmembrane domains (TMD1 to TMD7) and the calmodulin-binding domain (CaMBD) of the MLO protein were shown in solid boxes.

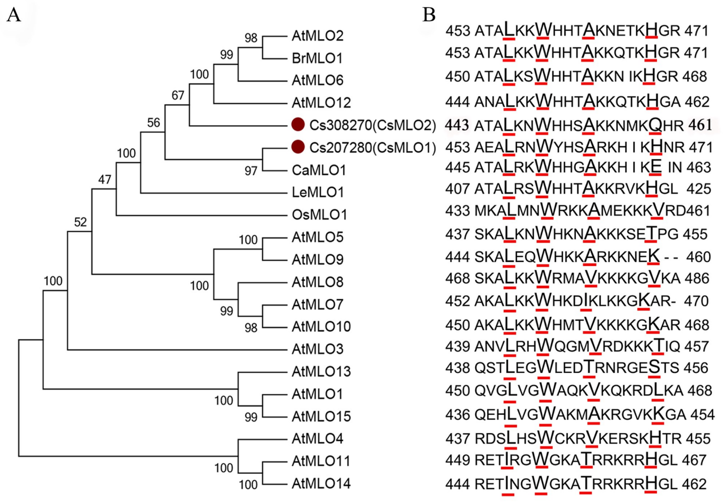

Figure S4. Phylogenetic analysis of CsMLO1/CsMLO2 and analysis of the CaM-binding domain (CaMBD) sequences of CsMLO1/CsMLO2. (**A**) Phylogenetic analysis of plant mildew resistance locus O (MLO) proteins. (**B**) Alignment of the putative CaMBD sequence of CsMLO1 (Alanine^{453} to Argine^{471}) and CsMLO2 (Alanine^{443} to Argine^{461}) with the corresponding regions of MLO families. The names of MLO families listed in (**A**) and their putative CaMBD sequences are aligned in the same order. The hydrophobic amino acids corresponding to a Ca^{2+}-dependent CaM-binding motif and the highly conserved tryptophan (W) residues were the capital letters.

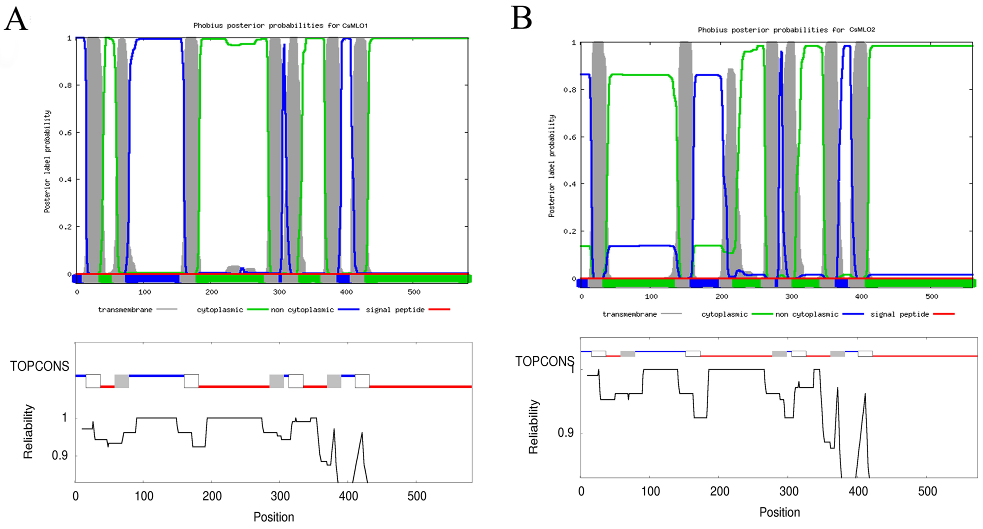

Figure S5. The predicted transmembrane topology of the CsMLO1 and CsMLO2 proteins analyzed. Cytoplasmic and non-cytoplasmic represented the intracellular and extracellular locations, respectively, using the online tools Phobius (http://phobius.sbc.su.se/) and TOPCONS (http://topcons.cbr.su.se/).

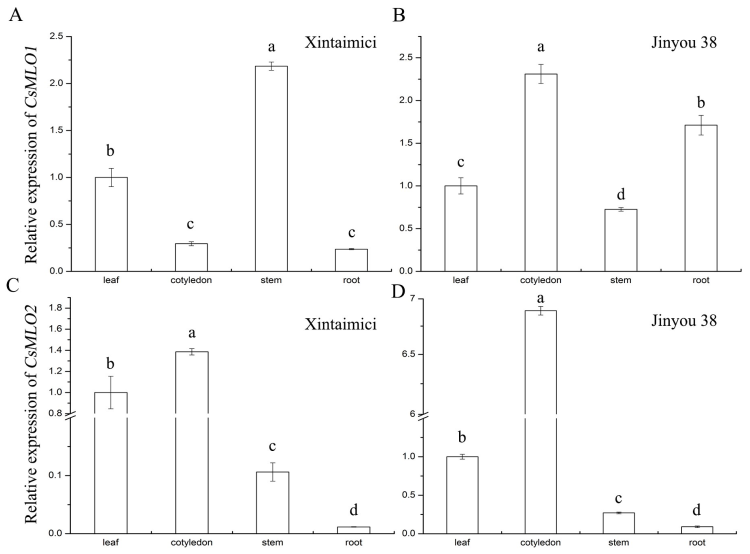

Figure S6. Expression pattern of *CsMLO1* and *CsMLO2* in cucumber. Spatial expression patterns of *CsMLO1* and *CsMLO2* in leaves, cotyledons, stems and roots according to reverse-transcription quantitative PCR (RT-qPCR). Expression analysis of candidate genes using the $2^{-\Delta\Delta Ct}$ method. Data are means ± standard deviations from three biological replicates per cultivar. The lowercase letters indicate significant differences according to Duncan's multiple range test (P < 0.05). (**A**) Expression pattern of *CsMLO1* in susceptible cultivar. (**B**) Expression pattern of *CsMLO1* in resistant cultivar. (**C**) Expression pattern of *CsMLO2* in susceptible cultivar. (**D**) Expression pattern of *CsMLO2* in resistant cultivar. The expression level in the leaf was normalized as 1. Data represent means ± SE of three biological replicates. Letters indicate significant differences at P < 0.05 compared with the leaf by Student's t-test.

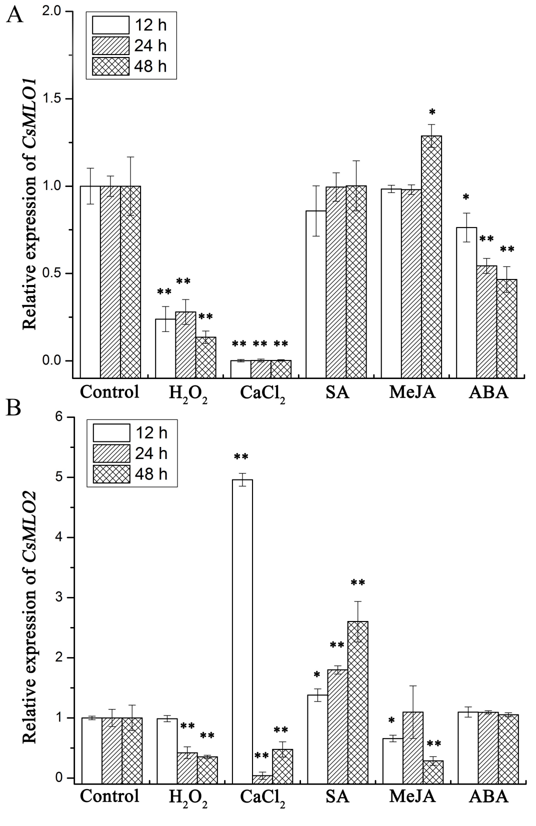

Figure S7. Expression patterns of *CsMLO1* and *CsMLO2* genes in response to plant hormones. (**A**) RT-qPCR analyses of *CsMLO1* transcripts in resistant variety under plant hormones treatments such as hydrogen peroxide (H_2O_2, 10 μM), abscisic acid (ABA, 100 μM), salicylic acid (SA, 1 mmol·L^{-1}), methyl jasmonate (MeJA, 100 μmol·L^{-1}) and $CaCl_2$ (10 mmol·L^{-1}). (**B**) RT-qPCR analyses of *CsMLO2* transcripts in resistant varieties under plant hormones treatments. The relative expression levels of *CsMLO1* and *CsMLO2* in cucumber plants at variety time points were compared with the mock control, which was set to 1. Expression analysis of candidate genes using the $2^{-\Delta\Delta Ct}$ method. Data are means ± standard deviations from three biological replicates per cultivar. The asterisks indicated a significant difference (Student's *t*-test, $*P < 0.05$ or $**P < 0.01$).
